# Toward a multi-level, needs-based approach to cancer navigation: Considerations for service design

**DOI:** 10.1007/s00520-026-10592-3

**Published:** 2026-03-25

**Authors:** Carla Thamm, Jaqueline Bender, Oluwaseyifunmi Andi Agbejule, Imogen Ramsey, Fiona Crawford-Williams, Carolyn Ee, Kristie McComb, Anurag K. Agrawal, Raymond J. Chan

**Affiliations:** 1 Caring Futures Institute, College of Nursing and Health Sciences, Flinders University, Adelaide, SA Australia; 2 Cancer Rehabilitation and Survivorship Program, Department of Supportive Care, Princess Margaret Cancer Centre, University Health Network, Toronto, ON Canada; 3 Dalla Lana School of Public Health and Institute of Health Policy, Management, and Evaluation, University of Toronto, Toronto, ON Canada; 4 American Cancer Society, Hagerstown, MD USA; 5 McGrath Foundation, Sydney, NSW Australia

## Abstract

Evidence suggests that patient navigation can help address ongoing barriers to accessing timely, appropriate, and quality cancer care. It also supports people affected by cancer to navigate through complex health care systems. Cancer patient navigation interventions include education, logistical, social, and emotional support, facilitating referrals, care coordination, patient advocacy, and enabling self-management, delivered by clinical and/or non-clinical navigators. For patient navigation services to be equitable and sustainable in resource-strained health care environments, there is a need to establish fit-for-purpose, flexible, multi-layered, innovative navigation programs. These programs must also integrate virtual care technologies and emerging innovations, such as artificial intelligence, to enhance service provision and reach. This commentary draws on a contemporary Australian framework for patient navigation and global practice standards to offer service design considerations. Further global efforts in research, practice, and policy development are crucial for building sustainable cancer patient navigation workforce models and ensuring high-quality service delivery.

## Introduction

Despite advances in cancer care, people affected by cancer experience barriers to accessing timely, quality care, including inadequate information provision, poorly coordinated care, and a lack of psychosocial, practical, financial, carer, and culturally appropriate support [1]. Patient navigation (PN) is critical to overcome these barriers and help people navigate complex health care systems at all stages of the cancer continuum [2, 3]. Effective PN programs are multidimensional and delivered by healthcare professionals and/or trained non-clinical navigators, who provide wide-ranging supports tailored to individuals’ needs [3] and spanning health, community, and social care systems [4, 5]. These programs are more established in North America and are being adapted and implemented globally, with ongoing efforts to expand and refine navigation service models. For services to be equitable and sustainable, navigation must move beyond isolated interventions, towards a collaborative, needs-based approach that acknowledges the complexity of each person’s experience [1, 6]. In this commentary, we offer service design considerations for multi-level, cross-sectoral, needs-based approaches to cancer PN.

## What is a needs-based approach to navigation?

A needs-based approach to cancer PN is a personalized and responsive way to address dynamic care needs, based on regular patient needs assessment, while acknowledging efficient use of limited resources [1]. It recognizes that everyone affected by cancer requires a foundational level of informational support (level 1); while some may desire/require additional supported navigation (level 2); or clinical navigation (level 3), with levels of support shifting throughout the cancer continuum in response to a person’s changing navigation needs [1].

### Level 1 navigation

All people affected by cancer, including carers and families, require information to navigate the cancer journey and access the care and support they need. [7, 8]. Information provision that supports patients and families to access cancer and supportive care could leverage digital solutions to offer a centralized resource hub with evidence-based information on cancer and a directory of supportive care interventions and services [1, 9]. A resource hub should be kept up-to-date and provide clear, trustworthy information that is easy to understand and available in multiple languages. While not directly providing navigation, a resource hub supports self-navigation and could be accessed with navigator support or programmed to recommend resources based on patient-reported need using recommender systems and AI-chatbot assistants.

### Level 2 navigation

An additional level of PN should be offered to people requiring extra support, extending beyond information provision to include psychosocial, financial, logistical and caregiver support, self-advocacy skills, and empowerment to help people navigate health and social care systems [1, 5, 6]. Navigation may be provided by non-clinical navigators, including lay/peer navigators (paid or volunteer). Peer navigators typically have lived experience with cancer and may also share other identities (e.g. diagnosis, gender, language, sexual orientation, cultural background). Peer navigators could be matched with patients through digital apps [8], and serve as trustworthy sources of experiential wisdom and support [10] to help reduce health care disparities [10]. While the value of peer navigators has long been recognized globally in community settings, these roles are increasingly being implemented in health care settings [11, 12]. Training and supervision of non-clinical navigators is critical to ensure the quality and effectiveness of PN services delivered [13, 14], and ensure program fidelity [15].

### Level 3 navigation

A more intensive level of PN should be offered to people when they are experiencing complex needs. Although PN should be offered to empower people affected by cancer to navigate health care, people affected by cancer may require navigation support, interventions, and advocacy to be carried out on their behalf at times. Interventions at this level could include direct clinical support, care coordination, and referrals to other health professionals or psychosocial services [1]. In many settings, specialist cancer nurses, nurse navigators, care coordinators, social workers or other trained clinical navigators would provide this level of navigation; however, some elements could also be delivered by non-clinical navigators depending on context, setting and navigation need. Innovative models should be developed through health services or other organisations, with a focus on regular assessment to recognise and respond to individual needs. Practice standards, such as those from the Professional Oncology Navigation Task Force [16] and the Global Initiative to Advance Cancer Navigation for Better Outcomes (GINO) [17], provide guidance for developing and implementing navigation models, tailored to the local context.

## What support is provided in needs-based navigation?

**Fig. 1:**
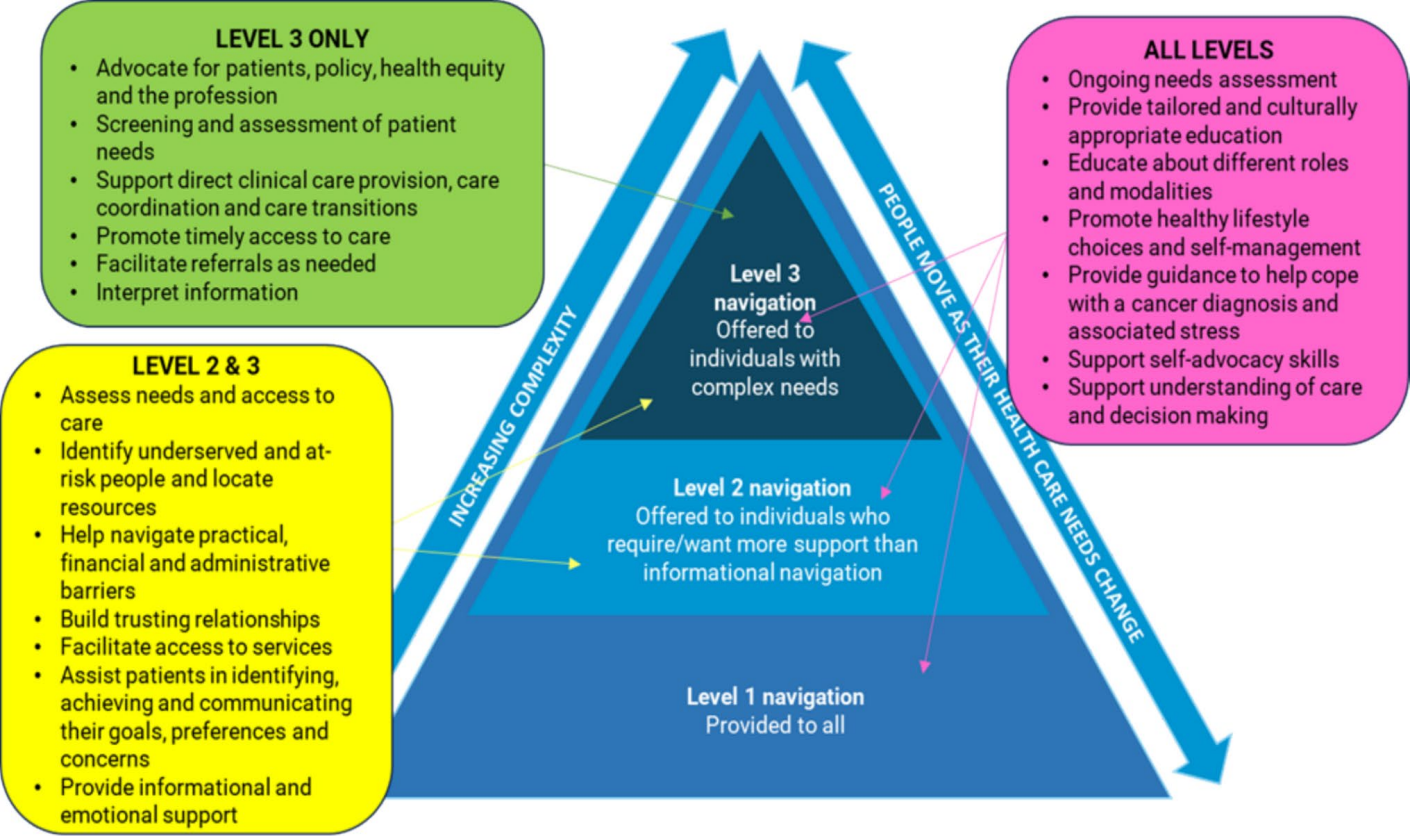
The Flinders needs-based patient navigation framework [18] mapped to GINO navigation practice standards [17]

The GINO practice framework [17] describes the scope of tasks or practices involved in providing PN in cancer. It recognizes that different settings may require varying navigator roles and levels of resourcing to support the implementation of navigation services. The framework classifies practices into the following domains: identifying and addressing barriers to care; care planning and coordination; communication; support of direct clinical care; education; psychological, social and emotional support; patient empowerment and advocacy [17]. Practices may be relevant to some or all the levels described in the needs-based framework [1]. In Fig. 1, we postulate how navigation practices may be considered in the context of a needs-based navigation framework [17, 18].

## Take-home messages

Applying a needs-based approach to PN could ensure timely, appropriate support given limited resources while enhancing efficiency, equity, and health care integration. To support adoption of PN services, it is essential to clearly define navigators’ required capabilities across practice domains and levels of PN. Establishing clear and transparent scopes–of practice for different roles [19] will enhance role clarity, standardization, and workforce sustainability. Additionally, competency-based navigator training is essential. A dynamic, needs-based approach to PN must also integrate virtual care technologies and emerging innovations, such as AI, to enhance services. Technology presents opportunities to streamline processes, improve communication, and expand reach, particularly in resource-limited settings. However, it is vital to ensure that digital solutions do not inadvertently widen disparities [20]. This proposed framework from Australia mapped to global practice standards provides a foundation for delivering PN services in cancer care but needs refining for the broader global context. Further research, practice, and policy development are crucial for building sustainable workforce models, fostering collaboration, integrating technology, and ensuring high-quality service delivery.

## Data Availability

No datasets were generated or analysed during the current study.
